# Using the pedicle screw-U rod system for the treatment of double-level lumbar spondylolysis and isthmic spondylolisthesis

**DOI:** 10.3389/fsurg.2024.1308389

**Published:** 2024-02-02

**Authors:** Jinghao Jiang, Tao Lin, Xia Chen, Rui Gao, Xuhui Zhou

**Affiliations:** ^1^ School of Health Science and Engineering, Shanghai University for Science and Technology, Shanghai, China; ^2^ Department of Orthopedics, Changzheng Hospital, Second Military Medical University, Shanghai, China

**Keywords:** pedicle screw-U rod system, range of motion, spondylolisthesis, spondylolysis, health-related quality of life

## Abstract

**Objective:**

The aim of this study was to evaluate the efficacy of the pedicle screw-U rod system in treating double-level lumbar spondylolysis with or without spondylolisthesis.

**Methods:**

A retrospective study was conducted. Twenty-six patients were included in this study and followed up at 3, 6, and 12 months. Patients without spondylolisthesis were treated with double U-shaped rods (group I), and patients with spondylolisthesis were treated with a lengthened U-shaped rod (group II). Japanese Orthopedic Association (JOA) scores, Oswestry disability index (ODI) scores, disc range of motion (ROM), intervertebral space height of fixed levels and adjacent levels, and grading the degeneration of adjacent segmental intervertebral discs were evaluated preoperatively and postoperatively.

**Results:**

JOA and ODI scores improved significantly at 3 months both in groups I and II. The average bone grafting healing time was 6.1 ± 3.1 months for group I and 6 ± 2.8 months for group II. The intervertebral space heights of L4/L5 and L5/S1 were improved significantly at the final follow-up (*p* < 0.05 for both groups). Surgical segmental and adjacent segmental ROM had no significant change at the final follow-up, in comparison with data preoperatively (*p* > 0.05). No significant changes of intervertebral space height (L3/L4) and grading of intervertebral disc degeneration were noted before and after surgery (*p* = 0.141 and 0.484, respectively).

**Conclusions:**

The pedicle screw-U rod system provided advantages of being easy in repairing symptomatic double-level lumbar spondylolysis. This technique improved disabilities of patients, preserved the lumbar spine ROM, and delayed the degeneration of adjacent segments.

## Introduction

Spondylolysis is defined as an anatomic defect in vertebral pars interarticularis. Multiple-level lumbar spondylolysis is rare and accounts for approximately 0.3% among the general population and involves two levels, L4 and L5, in more than 60% (1, 2). Spondylolisthesis refers to anterior or posterior slipping of one segment of the spine on the next lower segment, with the most common cause being isthmic. Generally, conservative treatment is recommended for most symptomatic patients with spondylolysis and/or spondylolisthesis (3). However, if patients do not respond to conservative measures, surgical intervention is usually indicated.

Various techniques have been advocated for the surgical management of spondylolysis or isthmic spondylolisthesis. Among these procedures, direct repair of the pars defect stabilized with a construct consisting of a pair of pedicle screws connected by a U-shaped rod has several advantages in achieving excellent bone graft healing, preventing anterior displacement of the diseased segment, and maintaining intervertebral space height (4–6). However, double-level spondylolysis and isthmic spondylolisthesis are rare, and it is difficult to define an optimal treatment algorithm for these. In this study, we describe a retrospective study evaluating the efficacy of the U-shaped rod and screws system for the treatment of double-level lumbar spondylolysis and isthmic spondylolisthesis, and to our knowledge, there is no similar research in the literature.

## Materials and methods

We retrospectively reviewed patients with double-level lumbar spondylolysis who were treated with a U-shaped rod and screws system at the Shanghai Changzheng Hospital between January 2014 and June 2016. This research was conducted in accordance with the tenets of the Declaration of Helsinki and was approved by the Institutional Review Board of the Changzheng Hospital. Written informed consents were obtained from all participants or their guardians prior to the study. The major inclusion criteria were as follows: (1) patients had symptomatic double-level bilateral spondylolysis at L4 and L5 with or without spondylolisthesis at L4 (Grade I‒IV, Meyerding criteria) (7); (2) patients presented with severe low back pain, with or without numbness of lower limbs and sciatica; (3) Grade I to II disc changes at L3 to S1 (Pfirrmann criteria) (8); (4) patients who had been resistant to conservative treatment for at least 6 months.

### Clinical evaluation

**Figure 1:**
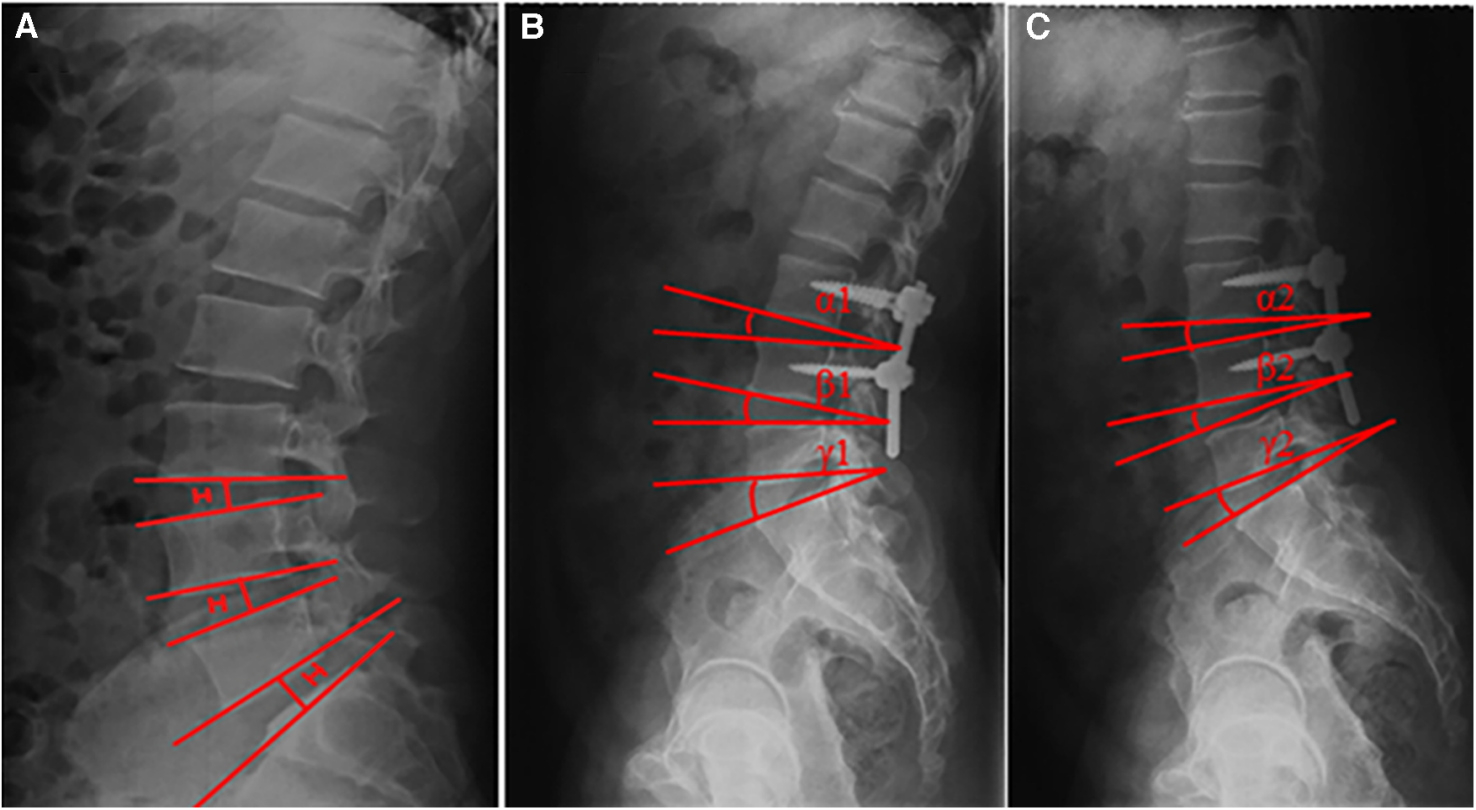
Measurement of intervertebral space height (**A**) and segmental ROM (**B,C**). The intervertebral space height (H) was determined by the distance between the midpoints of adjacent endplates of the vertebrae. Angles formed between the adjacent superior and inferior endplates were measured on flexion and extension images. The segmental ROM was determined by the difference between these two angles (i.e., *α*1–*α*2, *β*1–*β*2, and *γ*1–*γ*2).

Patients were followed up at 3, 6, and 12 months. The Japanese Orthopedic Association (JOA) score and the Oswestry disability index (ODI) score were used to evaluate lumbar disability preoperatively, postoperatively, and at the last follow-up. Lumbar magnetic resonance imaging (MRI) scan was performed preoperatively for all patients to evaluate the degree of disc degeneration below and above the defect. The images were graded using the Pfirrmann grading system for the assessment of lumbar disc degeneration (8). Anteroposterior, lateral, and flexion–extension radiographs, and lumbar computed tomography (CT) scan were performed to evaluate intervertebral space height (Figure 1A), segmental range of motion (ROM) (Figures 1B,C), and bone graft healing time independently by two radiologists preoperatively and at follow-up. Bone fusion was considered to be achieved if (1) trabecular bony bridge was formed at isthmuses, (2) the contact surface between the preoperative low-density area and bone graft became indistinct, and (3) the “dog neck sign” was indistinct.

### Surgical procedure

**Figure 2:**
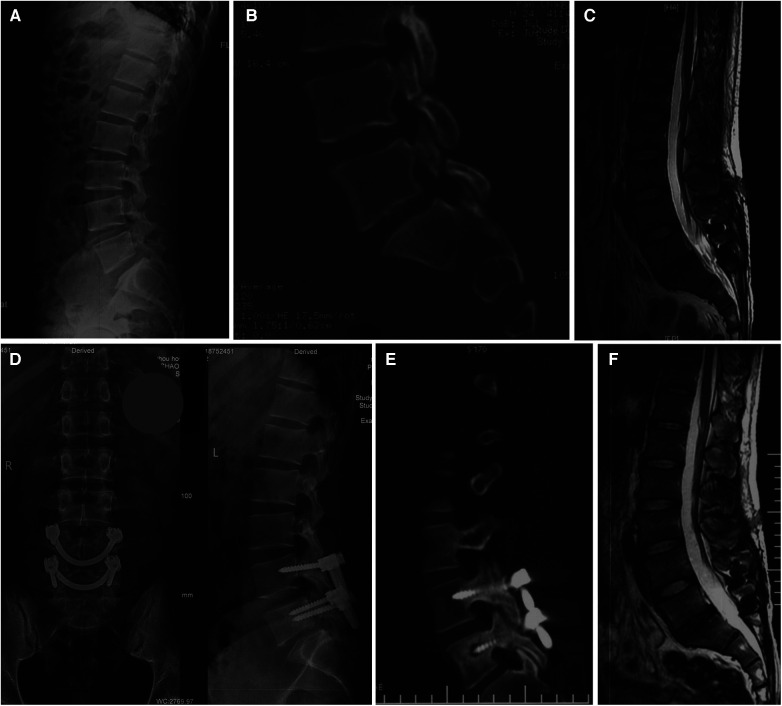
Preoperative and postoperative images obtained in a 26-year-old male diagnosed with double-level lumbar spondylolysis. (**A–C**) Spondylolysis at L4 and L5. (**D–F**) Postoperative radiographs.

Double-level spondylolysis without spondylolisthesis (group I) (Figure 2): The procedure was similar to those reported previously (4–6). The defect in the L5 pars was exposed, and cancellous bone autograft harvested from the iliac crest was placed in the defect and impacted. A universal pedicle screw was inserted into the pedicle on each side at L5. After that, a U-shaped rod was placed under the L5 spinous process and pushed to lock onto the pedicle screws. In this way, the rod was firmly fixed against the spinous process and the laminae, which promotes compression of the graft in the defect and stabilizes the posterior arch. The process was repeated for the second lytic vertebrae.

**Figure 3:**
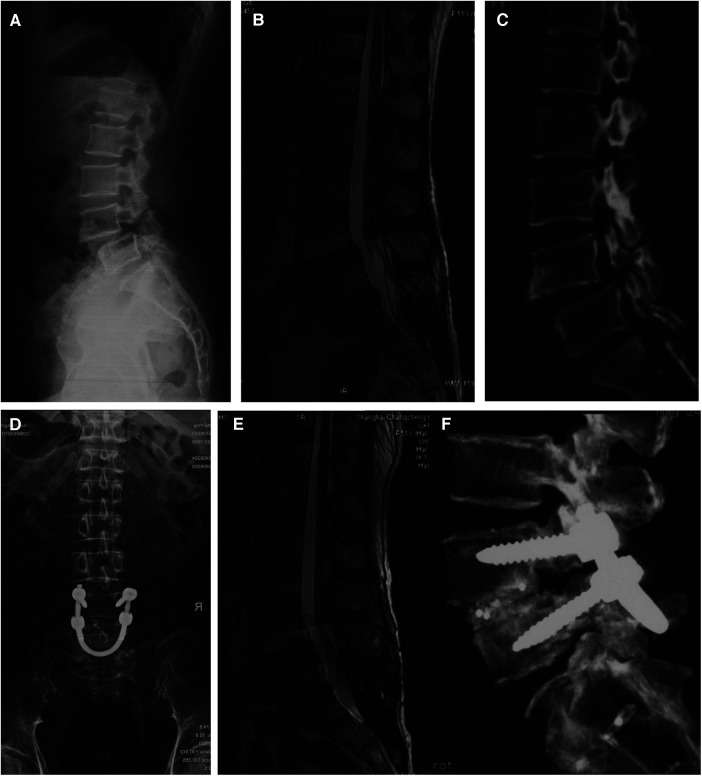
Preoperative and postoperative images obtained in a 51-year-old female diagnosed with double-level lumbar spondylolysis. (**A–C**) Spondylolysis at L4 and L5, and spondylolisthesis at L4. (**D–F**) Postoperative radiographs.

Double-level spondylolysis with spondylolisthesis (group II) (Figure 3): The defect in the L4 and L5 pars was exposed, and cancellous bone autograft harvested from the iliac crest was placed in the defect and impacted. A universal pedicle screw was inserted into the pedicle on each side at L4 and L5. Decompression was performed if necessary. A ring incision was made on one side of the L4/L5 annulus, and the disk material was removed with pituitary rongeurs. After removal of the disk, a U-shaped rod was placed under the L5 spinous process, and the arm of the rod was pushed to lock onto the pedicle screws and secured both sides. Finally, reduction was performed, and bone chips procured from the decompression was compressed into the disk space for bony fusion.

### Statistical analysis

Statistical analysis was performed using the SPSS 19.0 software (SPSS Inc., Chicago, IL). Differences in radiological parameters between two time points were compared using Student's *t*-test and the Wilcoxon signed-rank test. A two-tailed *p*-value <0.05 was considered to be statistically significant.

## Results

Twenty-six patients (21 men and 5 women) with double-level bilateral spondylolysis at L4 and L5 were eligible for the study. Their age ranged from 15 to 56 years, with an average of 35.7 years. Fifteen patients had Grade I or II spondylolisthesis at L4. Of the 26 patients, neurogenic symptoms were present in 10 patients, all of whom had spondylolisthesis. Patient demographic characteristics are shown in Table 1. No herniated lumbar disc was observed.

**Table 1:** Demographic characteristics of the patients.

	Group I	Group II
Sex	Female: 2 Male: 9	Female: 3 Male: 12
Age (years), mean ± SD	36.4 ± 1.5	35.0 ± 1.8
BMI (kg/m^2^), mean ± SD	23.4 ± 2.3	23.7 ± 2.1
Duration of conservative therapy (months), mean ± SD	3.8 ± 0.32	3.6 ± 0.28
Spondylolisthesis	NA	Grade I: 11 Grade II: 4
Presence of symptoms		
Low back pain	11	15
Sciatica	0	6
Numbness of lower limbs	0	4
EBL (ml), mean ± SD	117.4 ± 12.1	195.8 ± 13.2
ORT (h), mean ± SD	2.1 ± 1.1	2.2 ± 1.6
Bone healing time (months), mean ± SD	6.1 ± 3.1	6.0 ± 2.8
Duration of follow-up (months), mean ± SD	13.0 ± 1.3	12.7 ± 1.8

NA, not applicable; BMI, body mass index; EBL, estimated blood loss; ORT, operating room time.

**Figure 4:**
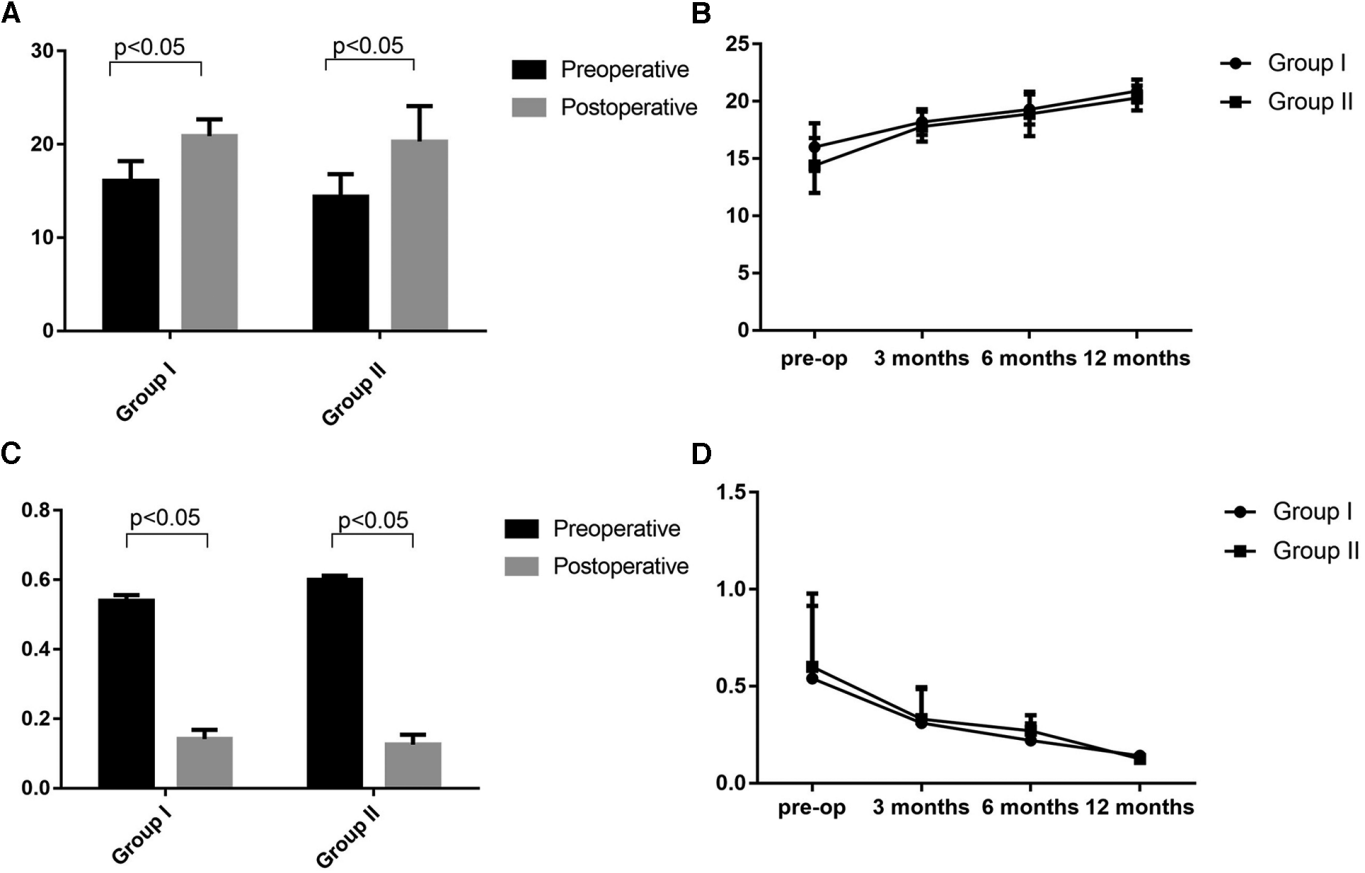
Preoperative and postoperative lumbar JOA (**A,B**) and ODI scores (**C,D**). Group I: double-level spondylolysis without spondylolisthesis. Group II: double-level spondylolysis with spondylolisthesis.

The patients were followed up for a mean duration of 12.9 ± 2.8 months. The JOA and ODI scores in both groups demonstrated statistically significant postoperative improvement. The mean JOA scores significantly improved from 16.0 ± 2.1 to 20.9 ± 1.8 in group I and from 14.4 ± 2.4 to 20.3 ± 3.8 in group II, respectively. Mean ODI scores improved significantly from 54% to 14.2% in group I and from 60% to 12.6% in group II (Figure 4). Sciatica and numbness of lower limbs improved effectively in the 10 patients. The average time of bone graft healing was 6.1 months in group I and 6.0 months in group II. Postoperative lumbar ROM had no significant differences with preoperative lumbar ROM at final follow-up in both groups (*p* > 0.05). The mean intervertebral space height of L4/L4 and L5/S1 levels increased from 14.1 ± 0.2 to 16.1 ± 0.2 mm and 14.2 ± 0.1 to 16.0 ± 0.2 mm (*p* < 0.001), respectively, after surgery in group I. The mean intervertebral space height of L4/L5 and L5/S1 levels changed from 13.9 ± 0.1 to 15.9 ± 0.2 mm and 14.0 ± 0.2 to 16.1 ± 0.1 mm mm (*p* < 0.001), respectively, in group II. The mean degree of listhesis (%) at the L4/L5 level in group II improved significantly at last follow-up (*p* < 0.0001) (Table 2). There were no signs of permanent nerve injury, breakage or loosening of the internal fixation, and other complications at the follow-up periods.

**Table 2:** Clinical and radiographic outcomes by group.

Parameters	Group I (*N* = 11)			Group II (*N* = 15)		
	Preoperative	Postoperative	*p*-value	Preoperative	Postoperative	*p*-value
JOA scores, mean ± SD	16.0 ± 2.1	20.9 ± 1.8	<0.0001	14.4 ± 2.4	20.3 ± 3.8	<0.0001
ODI scores, mean ± SD	54.0%	14.2%	<0.0001	60.0%	12.6%	<0.0001
Intervertebral space height (mm), mean ± SD	L3/L4 15.9 ± 0.2	L3/L4 16.0 ± 0.1	0.154	L3/L4 15.8 ± 0.1	L3/L4 15.9 ± 0.2	0.094
	L4/L5 14.1 ± 0.2	L4/L5 16.1 ± 0.2	<0.0001	L4/L5 13.9 ± 0.1	L4/L5 16.2 ± 0.2	<0.0001
	L5/S1 14.2 ± 0.1	L5/S1 16.0 ± 0.2	<0.0001	L5/S1 14.0 ± 0.2	L5/S1 16.1 ± 0.1	<0.0001
Spondylolisthesis						
Grade 0	11	11	—	0	11	<0.0001
Grade I	0	0	—	7	3	
Grade II	0	0	—	8	1	
Pfirrmann grading						
Superior level	Grade I: 8; Grade II: 3	Grade I: 8; Grade II: 3	—	I grade: 12; II grade: 3	—	—
Inferior level	Grade I: 9; Grade II: 2	Grade I: 9; Grade II: 2	—	Grade I: 13; Grade II: 2	Grade I: 13; Grade II: 2	—
ROM (°), mean ± SD						
L3/4	9.2 ± 0.6	9.1 ± 0.5	0.676	8.9 ± 0.2	8.8 ± 0.3	0.292
L4/5	9.7 ± 0.5	9.6 ± 0.2	0.545	—	—	—
L5/S1	10.1 ± 0.3	9.9 ± 0.4	0.200	10.1 ± 0.3	9.9 ± 0.5	0.195

Group I, double-level spondylolysis without spondylolisthesis; group II, double-level spondylolysis with isthmic spondylolisthesis.

## Discussion

The incidence of lumbar pars defect involving double levels is rare, with few reports of double-level lumbar spondylolysis in the literature. Double-level spondylolysis associated with spondylolisthesis is even rarer. The principle of double-level lumbar spondylolysis and spondylolisthesis treatment was the same as that for single-level spondylolisthesis (9). Direct pars defect repair with different kinds of internal fixation was adopted by different authors for treatment of symptomatic multiple-level lumbar spondylolysis (10–12), and they all achieved good results. However, these procedures had their own defects. A metal wire combined with bone grafting required prolonged immobilization with a lumbar brace, and a screw-hook combined with bone grafting was complicated and with a high risk of dura sac or nerves injury due to improperly positioned (13–16). Direct pars repair with the pedicle screw-U rod system is an alternative surgical procedure for multiple-level lumbar spondylolysis, providing stable fixation, excellent lumbar spine mobility, and improved JOA and ODI scores. In this study, we described the surgical outcome of 26 cases with double-level lumbar spondylolysis who were treated with the pedicle screw-U rod system.

It is reported that the incidence of multiple-level lumbar spondylolysis is higher in males (9, 17). Consistent with the trends reported in the literature, 21 of the 26 patients were male in our series. In this study, 15 had associated spondylolisthesis and most of them presented with neurogenic symptoms such as intermittent radiating leg pain and lower limb numbness. These patients underwent direct pars defect repair including placing the pedicle screws above and below the slip, securing the arms of the U rod at both sides following a posterior lumbar intervertebral fusion. For patients without spondylolisthesis, direct pars defect repair with the pedicle screw-U rod system was performed, just as reported previously (5). For these two groups, JOA and ODI scores improved significantly at the final follow-up postoperatively, and none of the patients complained of permanent low back pain, sciatica, or numbness of lower limbs. The rational for the pedicle screw-U rod system is to avoid the drawbacks of fusion, and the aim is to save a spinal motion segment to retain lumbar spine mobility and to restore normal anatomy. Ulibarri et al. evaluated the biomechanical property of the pedicle screw-U rod system in treating single-level spondylolysis and suggested that it provided excellent stability in interbody flexion–extension and torsion compared to the normal spine, which may be beneficial in maintaining adjacent-level motion and prevention of stress shielding (16). In the present study, there were no statistically significant differences in segmental lumbar ROM between preoperative and postoperative periods for both groups. Bone healing was achieved in all patients. These findings indicated that the pedicle screw-U rod system could effectively relieve the symptoms and provide rigid intrasegmental fixation with minimal intersegmental motion interference compared with traditional segmental fusion. Chen et al. showed similar results in treating single-level lumbar isthmic spondylolysis that there was no statistically significant difference in the ROM of the intervertebral disks before and after surgery (5).

Sairyo et al. conducted a three-dimensional finite element analysis, which demonstrated that lumbar spondylolysis increased disc stresses at the affected as well as cranial adjacent levels, and it might lead to disc degeneration at both levels, while direct pars defect repair could improve the biomechanical environment of diseased and adjacent intervertebral discs (18). In our study, increasing adjacent intervertebral degeneration was not observed in both groups at 12 months postoperatively. The surgical intervertebral space height was improved significantly (*p* < 0.05), and adjacent intervertebral space height had no differences (*p* > 0.05) before and after surgery. In the 15 patients with spondylolisthesis, there was no change in the spondylolisthesis grade at the latest follow-up. These findings showed that the pedicle screw-U rod system could provide enough force and stability to prevent a second slip of surgical segments and would not put extra burden on the intervertebral space discs both in surgical and adjacent segments.

The efficacy of direct pars defect repair in treating spondylolysis-associated spondylolisthesis still remains obscure. Mohi Eldin reported that direct pars defect repair was useful for fusion of the pars defect with a minimal degree of isthmic spondylolisthesis (19). Chen et al. (5) and Koptan et al. (20) both suggested that direct pars defect repair was applicable to spondylolysis with grade I spondylolisthesis. However, several authors thought that in cases with an isthmic slip of over 25%, the affected motion segment would not be biomechanically normal even after a successful bony healing of the pars defect. Liu et al. demonstrated that multiple lumbar spondylolysis with spondylolisthesis could not be treated by direct pars defect repair as this procedure achieved inadequate segmental stability (9). In the present research, we used the pedicle screw-U rod system to fix L4 and L5 spondylolysis with L4 spondylolisthesis, without considering the level of vertebral slip. A lengthened U-shaped rod was passed beneath the spinous process of L5 and was locked onto pedicle screws at L4 and L5. Intervertebral fusion was also performed at the L4/L5 level. The bone chips procured during posterior decompression were used as interbody grafts instead of a cage, which showed a similar result as in the literature (21). This procedure would effectively restore L4 to normal position and decrease the forward shear stress on L4. Although this procedure sacrificed the mobility of the L4/L5 segment, it preserved the mobility of L3/L4 and L5/S1 segments (*p* > 0.05, compared with preoperative mobility). At the latest follow-up, none of the patients had a second spondylolisthesis on L4, and the JOA and ODI scores improved significantly at 12 months postoperatively.

However, several limitations could not be ignored. First, this was a retrospective study conducted at a single institution, and the relative small sample size limited the power of our analysis. Second, no control groups were set, which might reduce the reliability of the research. Third, a mean of 12 months of follow-up might be a little short to evaluate the efficiency of the surgery.

## Conclusion

The pedicle screw-U rod system provided significant improvements in directly repairing symptomatic double-level lumbar pars defects with or without spondylolisthesis. This technique showed improvements in low back pain, sciatica, and numbness of lower limbs in patients with spondylolysis and spondylolisthesis. It could also preserve the ROM of lumbar spine and would not aggravate the degeneration of adjacent segments.

## Data Availability

The original contributions presented in the study are included in the article/Supplementary Material, further inquiries can be directed to the corresponding authors.
